# Ramping single unit activity in the medial prefrontal cortex and ventral striatum reflects the onset of waiting but not imminent impulsive actions

**DOI:** 10.1111/ejn.12895

**Published:** 2015-04-20

**Authors:** Nicholas A Donnelly, Ole Paulsen, Trevor W Robbins, Jeffrey W Dalley

**Affiliations:** 1 Behavioural and Clinical Neuroscience Institute, University of CambridgeCambridge, UK; 2 Department of Psychology, University of CambridgeDowning Street, Cambridge, CB2 3EB, UK; 3 Department of PDN, University of CambridgeCambridge, UK; 4 Department of Psychiatry, University of CambridgeCambridge, UK

**Keywords:** impulsivity, neurophysiology, nucleus accumbens, rat, visual attention

## Abstract

The medial prefrontal cortex (mPFC) and ventral striatum (VS), including the nucleus accumbens, are key forebrain regions involved in regulating behaviour for future rewards. Dysfunction of these regions can result in impulsivity, characterized by actions that are mistimed and executed without due consideration of their consequences. Here we recorded the activity of single neurons in the mPFC and VS of 16 rats during performance on a five-choice serial reaction time task of sustained visual attention and impulsivity. Impulsive responses were assessed by the number of premature responses made before target stimuli were presented. We found that the majority of cells signalled trial outcome after an action was made (both rewarded and unrewarded). Positive and negative ramping activity was a feature of population activity in the mPFC and VS (49.5 and 50.4% of cells, respectively). This delay-related activity increased at the same rate and reached the same maximum (or minimum) for trials terminated by either correct or premature responses. However, on premature trials, the ramping activity started earlier and coincided with shorter latencies to begin waiting. For all trial types the pattern of ramping activity was unchanged when the pre-stimulus delay period was made variable. Thus, premature responses may result from a failure in the timing of the initiation of a waiting process, combined with a reduced reliance on external sensory cues, rather than a primary failure in delay activity. Our findings further show that the neural locus of this aberrant timing signal may emanate from structures outside the mPFC and VS.

## Introduction

Impulsivity describes the tendency to make rapid decisions and actions without planning (Evenden, 1999; Winstanley *et al*., 2006). It is often present in attention deficit hyperactivity disorder (Solanto *et al*., 2001; Sonuga-Barke, 2003) and may play a causal role in drug addiction (Jentsch & Taylor, 1999; Dalley *et al*., 2011). Neurally, impulsive actions and decisions are widely hypothesized to originate from impaired connectivity and function of the prefrontal cortex and subcortical structures involved in the timing and control of goal-directed behaviour (Winstanley *et al*., 2006; Dalley *et al*., 2011; Kim & Lee, 2011; Sato *et al*., 2012; Jupp *et al*., 2013; Merchant *et al*., 2013; Simon *et al*., 2013; Hayes *et al*., 2014). Lesions of the medial prefrontal cortex (mPFC) and ventral striatum (VS), including the core and shell of the nucleus accumbens, are widely reported to disrupt several forms of impulsivity in rodents, including the timing of responses for delayed rewards (Pothuizen *et al*., 2005), anticipatory responding (Robbins, 2002; Christakou *et al*., 2004), and discounting of delayed and probabilistic rewards (Cardinal *et al*., 2001; Acheson *et al*., 2006; Cardinal, 2006; Valencia-Torres *et al*., 2012).

However, few studies have investigated the relationship between single neuron activity in these candidate brain regions and impulsivity. The mPFC has been implicated in a wide range of cognitive control processes including working memory, attention, action–outcome learning, reward, and conflict/error monitoring (Miller, 2000; Fuster, 2001; Euston *et al*., 2012). A common function proposed for the mPFC is the temporal representation of behavioural sequences (Bekolay *et al*., 2014). Such activity may underlie waiting processes (Ollman & Billington, 1972; MacDonald & Meck, 2004), internal representations of time (Jin *et al*., 2009; Kim *et al*., 2013), decision-making (Usher & McClelland, 2001; Roitman & Shadlen, 2002; Gold & Shadlen, 2007), response value or action restraint (Narayanan & Laubach, 2009; Jimura *et al*., 2013) and the co-ordination of sequences of actions (Ma *et al*., 2014).

The VS has been described as a ‘limbic motor interface’ (Mogenson *et al*., 1980) linking motivation to the selection (Nicola, 2006; Humphries & Prescott, 2010) and invigoration (McGinty *et al*., 2013) of behavioural responses. Single unit activity in the VS has been particularly linked to reward and reward-predictive cues (Shibata *et al*., 2001; Cromwell & Schultz, 2003; Setlow *et al*., 2003; Ishikawa *et al*., 2008; Van der Meer & Redish, 2009; Lansink *et al*., 2012; McGinty *et al*., 2013) and there is evidence that VS representations of reward or task outcomes are dynamic and prolonged throughout delays (Lavoie & Mizumori, 1994; Miyazaki *et al*., 1998; Van der Meer & Redish, 2009; Van der Meer *et al*., 2010; Jimura *et al*., 2013).

In the present study we used a multi-channel microelectrode system to record the extracellular activity of single units in the mPFC and VS of rats performing a visual attention task. The five-choice serial reaction time task (5-CSRTT) assesses attentional control over behaviour and the capacity to suppress a pre-potent response until the onset of a visual target stimulus (Robbins, 2002). We specifically investigated whether neural activity in these regions is modulated by the delay period leading up to a response, when response inhibition is required, and whether this activity differs between the mPFC and VS in predicting an upcoming premature response.

## Materials and methods

### Subjects

Male Lister-Hooded rats (mPFC, *n* = 8; VS, *n* = 8; Charles River, UK) were trained on the 5-CSRTT following the method of Bari *et al*. (2008). Throughout all of the experiments rats were housed under a reversed light cycle at 20 °C, with white lights off (red lights on) between 07:00 and 19:00 h. Rats were food deprived to 85% of free feeding weight (17–19 g of standard laboratory rat chow per day), and allowed access to water *ad libitum*. Rats were housed in groups of four during training, but after surgery were singly housed to prevent cage-mates from damaging their implants. All experimental procedures were carried out in accordance with the UK Animals (Scientific Procedures) Act of 1986 and the Council Directive 2010/63EU of the European Parliament on the protection of animals used for scientific purposes, and approved by local ethical review at the University of Cambridge.

### Behavioural apparatus and training

**FIG. 1:**
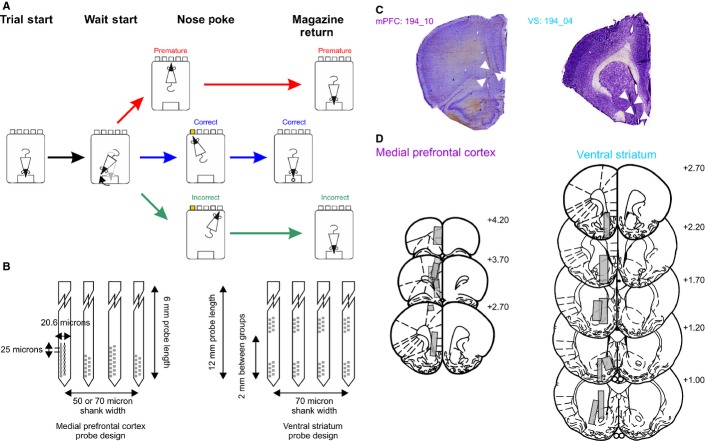
Task configuration and histological verification of microelectrode tracks. (A) Schematic of the 5-CSRTT and electrophysiological alignment events. Correct (blue), incorrect (green) and premature (red) response trials are shown in different colours, which are consistently used throughout the remaining figures. Trials began with a nose-poke response in the food magazine (Trial Start), which initiated a delay period of 5 s. In order to make a nose-poke response, rats must leave the food magazine and turn to face the nose-poke apertures (Wait-Start). Rats then either wait successfully for the delay period, after which time a 0.5 s light stimulus is presented in one of the nose-poke apertures, or fail to wait and nose-poke before the stimulus light (Premature responses). A response to the illuminated nose-poke aperture within 5 s is deemed to be a correct response and is rewarded with a food pellet in the food magazine. Premature and incorrect responses (where the rat successfully waits until the end of the delay period, but pokes in a non-illuminated aperture) lead to a 5 s time-out period during which time a new trial cannot be started and no food pellet is dispensed. (B) Schematic representation of the silicon probe design used in the mPFC (left) and VS (right) recordings. (C) Example of microelectrode tracks in the mPFC (left) and VS (right) with probe tracks highlighted with white arrowheads. (D) Reconstructed electrode tracks projected onto coronal sections of the rat brain (Paxinos & Watson, 2007) for the mPFC (left column) and VS (right column). Electrode placements were reconstructed from the most ventral point electrode tracks were observed and total distance travelled was estimated by the total number of drive turns made during the recording sessions. Numbers indicate the position of the sections relative to bregma (mm).

The 5-CSRTT is widely used to assess selective, spatially divided attention and response inhibition during a pre-defined waiting period (Robbins, 2002). Rats were trained in an operant chamber (Med Associates, VT, USA) to wait for a fixed delay (5 s), before the brief presentation of a light stimulus (0.5 s) in one of five nose-poke apertures, arranged in a curved array, which signalled to the rat in which aperture to make a nose-poke response (Fig.1A). Nose-poke responses within a fixed time window (the limited hold, 5 s) were reinforced with the delivery of a food pellet (45 mg Noyes Dustless pellets; Sandown Scientific) to a food magazine located on the opposite wall of the chamber to the nose-poke apertures, whereas responses before the delay had elapsed (premature responses) or in the wrong hole after a stimulus had been presented (incorrect responses) were punished with a 5 s time-out period whereupon all lights in the operant chamber were extinguished and the rat was unable to start a new trial. Similarly, a failure to make a response (omission responses) was punished with a 5 s time-out period. The end of the time-out was signalled by the house light and a light in the food magazine being reilluminated, after which time a new trial could be initiated by a nose-poke in the food magazine. A behavioural session finished when either a total of 100 trials (not including premature trials) were completed, or 30 min had elapsed. Behavioural experiments were controlled by a PC running Whisker software (Cardinal & Aitken, 2010), with the 5-CSRTT controlled via a custom-written matlab program.

### Microelectrode implantation

Rats of 7 months of age were implanted with either a single four-shank 54-channel silicon probe targeting the left mPFC or a single four-shank 64-channel silicon probe targeting the left VS. The left hemisphere was targeted as delay activity has been reported in the mPFC bilaterally (Narayanan & Laubach, 2006; Kim *et al*., 2013), but the left nucleus accumbens core has been particularly implicated in phenotypic high levels of premature responding in the 5-CSRTT (Caprioli *et al*., 2014).

All surgeries were carried out using aseptic technique under isoflurane anaesthesia (Isoflo; Abbott Laboratories, UK), using standard small animal stereotactic methods. Anaesthesia was induced with 4% isoflurane in 4 L/min O_2_, and maintained with 2% isoflurane in 2 L/min O_2_ delivered through a nose-cone. Peri-operative analgesia [Carprofen (Rimadyl; Pfizer), 5 mg subcutaneous injection] and prophylactic antibiotics [Enorfloxacin (Baytril; Bayer), 2.5% oral solution, 1 mL/100 mL drinking water)] were provided. All stereotactic measurements were made relative to bregma in the flat-skull position.

For mPFC probes, the shanks were 6 mm in length, either 50 or 70 μm in width (shanks alternated between 50 and 70 μm) and 15 μm in thickness. Shanks of 50 μm width had 11 electrodes, whereas shanks of 70 μm width had 16 electrodes, located at the tip of the shank in two rows. The electrode contact centres were spaced 20.6 μm apart horizontally, and 25 μm apart vertically. For recordings in the VS, 64-channel silicon probes were used (length 12 mm, width 70 μm, 35 μm thickness, and 16 electrode contacts per shank). The electrode geometry was the same as the mPFC electrodes, but on each shank electrodes were split into two groups of eight, with one group being at the tip of the electrode, and the second group 2 mm higher up the shank (Fig.1B).

Probes were implanted at the following co-ordinates relative to bregma (in mm): mPFC: anterior/posterior +2.7, medial/lateral +0.5, dorsal/ventral −3.0; and VS: anterior/posterior +1.7, medial/lateral +1.9, dorsal/ventral −6.0 (Paxinos & Watson, 2007). The final locations of the probes are shown in Fig.1C and D. A craniotomy opened over the mPFC or VS and a durotomy was performed. The probe, mounted on a stainless steel microdrive, was lowered into the brain to the appropriate depth. Implants were anchored to the skull with two T-shaped bolts inserted bilaterally in the parietal bone with a further bolt over the midline cerebellum, posterior to lambda, acting as an additional anchor and also as the recording ground. The microdrive assembly was secured to the bolts and the implant was encapsulated with dental acrylic (Simplex Rapid, Kemdent, UK).

At the end of the experiments, rats were overdosed with 1.5 mL sodium pentobarbital (200 mg/mL; Dolethal, Vetoquinol, UK), and perfused transcardically with 4% neutral buffered formalin. Brains were removed and cryoprotected in 30% sucrose prior to being sectioned on a freezing microtome (60 μm thickness), mounted on gelatin-subbed glass slides and stained with cresyl violet (Fig.1C and D).

### Electrophysiological recordings

Neurophysiological and movement data were recorded using a wireless 64-channel recording system (Triangle Biosystems, NC, USA), running Neuroware data acquisition software and Optimap online video-tracking. Neurophysiological signals were sampled at 30 kHz, and movement data were extracted from tracking two coloured LEDs located on the recording headstage, sampled at 30 Hz, using a Logitech C270 webcam. During the 5-CSRTT rats must wait for 5 s after the initiation of a trial before a visual stimulus is presented. During this waiting period rats typically leave the food magazine in advance of the time of stimulus presentation and engage in ‘scanning’ behaviour, where they look at the stimulus lights in turn, scanning their heads between each aperture (Humby *et al*., 1999; Robbins, 2002; Blondeau & Dellu-Hagedorn, 2007). The start of waiting (‘wait-start’) was defined as the time that rats left the food magazine, as measured using video tracking (Donnelly *et al*., 2014).

For some behavioural sessions the delay period on each trial was varied pseudorandomly between 4, 5, 6, 7 or 8 s (uniformly distributed). These sessions were longer than standard 5-CSRTT recording sessions (1 h, compared with 30 min), and the maximum number of trials was increased from 100 to 200 trials (not including premature responses), in order to capture sufficient responses at each delay for analysis. A total of 56 standard 5-CSRTT recording sessions (including 5390 trials with 4061 correct responses, 861 incorrect responses and 468 premature responses) and seven variable delay sessions (including 1151 trials with 620 correct responses, 194 incorrect responses and 337 premature responses) were analysed in mPFC-implanted rats, and 65 standard 5-CSRTT recording sessions (including 6036 trials with 4774 correct responses, 939 incorrect responses and 323 premature responses) were analysed in VS-implanted rats.

After each recording session, microdrives were advanced by at least 125 μm. All spikes included in the analysis were recorded from electrodes that passed through the mPFC or VS on sessions where the electrodes were located in those structures. This was based on the number of drive turns made and the estimated final co-ordinates of the electrodes derived from histological examination. Units were collectively analysed from sites throughout the regions of the mPFC and VS rather than making distinctions between either the dorsal and ventral PFC or the core and shell of the nucleus accumbens. We pooled cells in this way because it was difficult to define the precise location of recording electrodes on each particular recording session.

### Data analysis

All data analysis was performed using custom written-matlab (The Mathworks, Natick, MA, USA) and r (R Development Core Team, 2009) scripts. Single unit activity was extracted from raw signals using the Klusta suite of software tools (http://klusta-team.github.io/index.html; Kadir *et al*., 2014; Rossant *et al*., 2015). Raw data sampled at 30 kHz had their common median subtracted to remove common noise (Rolston *et al*., 2009) and were input into Spikedetekt. Features extracted from each detected spike were then clustered using Masked Klustakwik. Clusters were manually refined using Klustaviewa software.

Peri-stimulus spike rate histograms (PSTHs) were calculated relative to four task alignment events (Fig.1A): trial start, wait-start, nose-poking and magazine return. These alignment events were selected in order to capture the key behavioural events occurring in a 5-CSRTT trial: the nose-poke in the food magazine that initiates a trial, the self-paced start of waiting/'scanning’ behaviour, the time of behavioural responding, and the return of the rat to the food magazine, either to receive a food reward, or to terminate a post-error time-out period. We have previously demonstrated that the movement parameters of rats at the wait-start and nose-poke events are highly consistent, and particularly that the nose-poke event is consistently preceded by a discrete initiation of movement on all trial types (Donnelly *et al*., 2014).

**FIG. 2:**
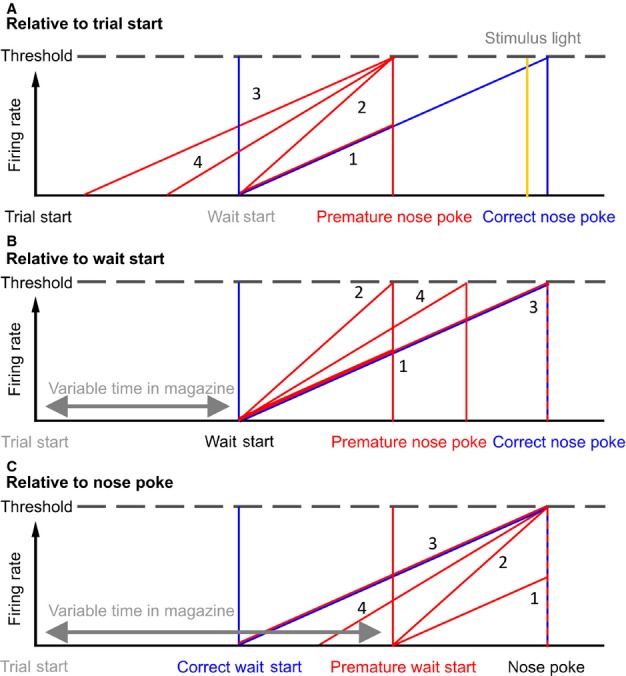
Putative models of ramping activity in relation to behaviour. (A) Possible models of delay activity in correct and premature responses on the 5-CSRTT. Proposed firing rate models relative to the start of a trial. Ramping firing rates for correct and premature trials are represented as diagonal lines, with the time of events as vertical lines, colour coded for trial outcome type. The time of the stimulus light is indicated as a vertical yellow line. Numbers indicate the firing rate model associated with the pattern illustrated (see Results). (B) The same proposed firing rate models relative to the start of waiting. (C) Proposed firing rate models relative to nose-poke responses. Note that this figure uses the example of firing rates increasing towards a threshold, but it would be equally valid to consider firing rates decreasing from some tonic level.

The PSTHs were estimated using two methods. For the illustrative examples shown in Fig.2A and B, the PSTH was calculated using Bayesian adaptive regression splines (Wallstrom *et al*., 2008). However, the Bayesian adaptive regression splines method requires multiple trials to give an effective estimate of the PSTH. For quantitative analysis of spike firing rates where it was necessary to have an estimate of firing rates on each trial, PSTHs were calculated by binning spike times into 1 ms bins, and convolving the binned data with Gaussian kernels (a kernel SD of 100 ms was used). The firing rates were then converted to *z*-scores to allow comparison between cells by subtracting the mean firing rate of each cell over the whole session, and dividing by the SD of the cell's firing rate over the whole session. Mean and SD firing rates over the whole session were calculated as the mean and SD of the reciprocal of all inter-spike intervals. In order to generate a dataset with sufficient spikes in peri-event windows for meaningful analysis, cells with average firing rates of < 0.5 spikes/s were excluded. Additionally, during some sessions, rats made very small numbers of premature responses. For sessions with < 4 premature trials, only correct and incorrect trials were analysed.

Principal components analysis (PCA) was performed using the matlab function *PCA*. The average PSTH from correct trials was calculated for all cells, and combined in a matrix of dimensions ([number of time bins] × [number of cells]), which was then used for PCA, giving [number of time bins] principal components (PCs). The same PCs were extracted from the average incorrect and premature trials for all cells by matrix multiplication of the average PSTH for incorrect and premature responses for each cell with the PCA coefficients.

Cells that were predictive of trial outcome were identified by first extracting features from PSTH windows using PCA; here the first three PCs were used. These components were then used as independent variables in a logistic regression model (fit using *fitglm* in matlab with a binomial distribution) to predict the outcome of the upcoming trial. Model classification performance was estimated using the area under the receiver-operator characteristic curve (AUC), calculated after leave-one-out cross-validation, where the model was fit to every trial but one, and the model was then used to predict the outcome of the left-out trial. The predicted and true trial outcomes were then used to calculate the AUC using *perfcurve* in matlab. The AUC was calculated for firing rates in a 1 s window, advanced in steps of 0.1 s for all cells. Example cells were identified from cells with AUC > 0.5 (i.e. cells where the firing rate features were able to provide above-chance classification performance) in the window from 1 s before to the time of nose-poking.

Delay-ramping neurons were identified as cells meeting a criterion of a significant linear regression (*P* < 0.05/[number of cells]) between the average firing rate on correct trials and time in the 3 s prior to nose-poking, in PSTHs calculated relative to the time of nose-poking, with a ¦*r*¦-value > 0.5 (where *r* is the Pearson product-moment correlation coefficient, from matlab function *corr*) and a ¦β¦ value > 0.3 (where beta is the gradient of the least-squares regression line, from matlab function *polyfit*). Positive ramping cells were identified as having a positive beta value, whereas negative ramping cells had negative beta values.

The variables influencing the PSTH at each point in time were estimated using a general linear model (GLM), fit using *fitglm* in matlab, with firing rate as the dependent variable. GLMs were fit to each time bin (100 ms bin size) for each cell, and the proportion of cells with significant effects of the independent variables was tested for significance (whether there were more cells with significant effects of a factor than would be expected by chance) using a binomial test, similar to the method of Schmidt *et al*. (2013). The threshold for significance used was 0.05/[number of time bins], in order to correct for multiple comparisons.

## Results

In total, 897 cells with an average firing rate of > 0.5 Hz were recorded in the mPFC, whereas in the VS the activity of 383 cells was recorded (see Fig.1C and D for final electrode positions). Rats with electrodes implanted in the mPFC and VS did not differ in their behavioural performance over the course of the recording experiments (all *P* > 0.05 for % accuracy, % correct response, % omissions, % premature, and correct response and reward collection latencies, Table 1).

**Table 1:** Behavioural data

	Mean	SD	*T*	*P*
% Correct				
mPFC	75.09	13.32	0.025	0.876
VS	74.53	10.13		
% Accuracy				
mPFC	81.63	10.05	0.319	0.581
VS	83.16	7.21		
% Omissions				
mPFC	8.61	8.11	0.096	0.761
VS	10.61	7.75		
% Premature				
mPFC	9.21	12.40	2.641	0.126
VS	5.07	5.55		
Correct latency				
mPFC	0.56	0.09	4.043	0.064
VS	0.64	0.16		
Collection latency				
mPFC	1.19	0.18	1.420	0.253
VS	1.26	0.21		

Values were taken over whole behavioural recording sessions (mPFC, *n* = 56; VS, *n* = 64). % Correct = no. of correct responses (*C*)/(*C* + no. of incorrect responses (*I*) + no. of omission responses (*O*)); % Accuracy = *C*/(*C* + *I*); % Omissions = *O*/(*C* + *I* + *O*); % Premature = no. of premature responses/(*C* + *I* + *O*). Correct latency, the average time (in seconds) between the illumination of the cue light and correct nose-poke responses; collection latency, the average time (in seconds) between correct nose-poke responses, and the rat returning to the food magazine to collect a reward pellet. *T*, *t* statistic for test for difference between mPFC and VS groups; *P*, p statistic for the same test.

Four hypotheses were investigated to determine whether there was ramping or delay activity during the waiting period of the 5-CSRTT, which could be used to predict upcoming impulsive responses. (1) Premature nose-poke responses occur earlier than correct responses, so ramping activity reaches a lower level at the point when nose-poking occurs than that reached on correct trials. This relationship assumes that the firing rate increase has the same gradient on correct and premature trials, and that ramping starts at similar times for each trial. However, the occurrence of behavioural responding is determined by some process occurring in addition to ramping activity; if the triggering of a response was related to ramping reaching a threshold then premature responses could not be explained by this model. (2) Premature responses occur when firing rates reach a threshold, and ramping activity has a steeper gradient until that threshold is reached than on correct trials. In both models (1) and (2), the firing rate differences occur despite the rats starting to wait (i.e. leaving the food magazine to engage in ‘scanning behaviour’) at a similar time to trials ending in correct responses. (3) Premature responses follow the same ramping gradient as correct responses but rats start the waiting process earlier by leaving the food magazine earlier. As in model (2) this model assumes that firing rates reaching a threshold value are involved in triggering a response. (4) A hybrid model – the rat leaves the magazine earlier, and also waits for less time than when a correct response occurs, so having a slightly steeper gradient in firing rate ramping than correct responses. These possible models are illustrated in Fig.2A–C, showing the model predictions as patterns of PSTHs relative to the start of a trial and the wait-start and nose-poke events. We hypothesized that delay-related single unit activity would begin at the onset of waiting, based on our previous finding that changes in power in the local field potential (LFP) gamma and theta frequency bands occurred at this event and, in the case of the theta band, spanned the period between wait-start and nose-poking (Donnelly *et al*., 2014).

### Cells predictive of premature responses

**FIG. 3:**
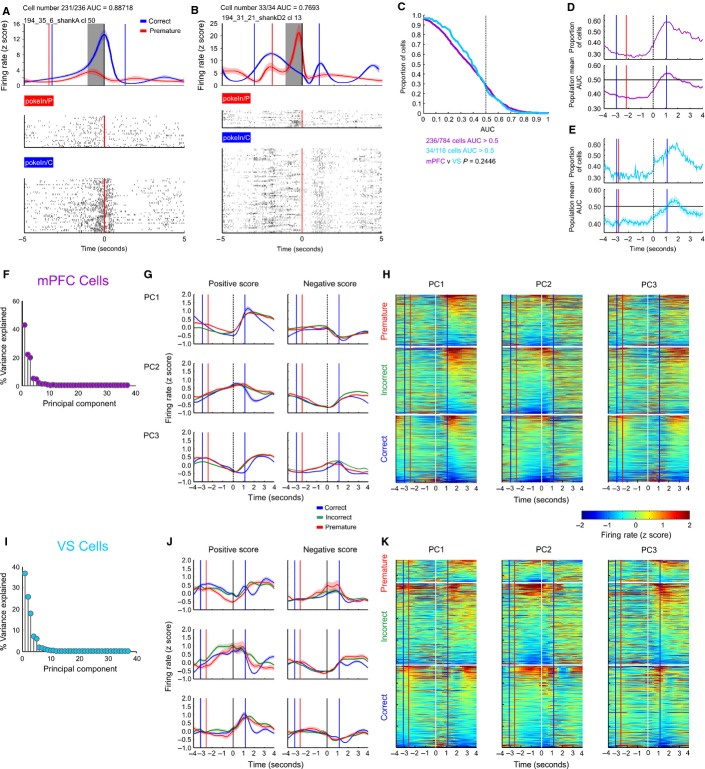
Trial outcome-predictive cells and population PCA. (A) Representative neuron recorded in the mPFC. PSTH aligned to nose-poking response for correct and premature responses (top). Coloured vertical lines indicate the median time to the preceding (Wait-Start, to left of time = 0) or subsequent (Return to the food magazine, to right of time = 0) behavioural event, for each outcome. Raster plots for all trial types (bottom) are ordered by time in the recording session from bottom to top. (B) Representative neuron recorded in the VS. (C) Distribution of AUC scores (a measure of predictive accuracy) in the window at 1 s before nose-poking for all cells recorded in the mPFC (magenta) and VS (light blue). The distribution of AUC scores did not differ between the mPFC and VS (Wilcoxon Rank-Sum test, *P* = 0.024). (D) Top – proportion of mPFC cells with AUC > 0.5 in a 1 s wide moving window around the nose-poke event. Bottom – mean of moving window AUC scores over the whole population of mPFC cells. Shaded area indicates SEM. (E) As D, for cells recorded from the VS. (F) Proportion of variance explained by all PCs calculated from peri-nose-poke firing rates of all cells recorded in the mPFC. (G) Average firing rate of cells with a positive or negative score on the first three PCs, with the population average of correct, incorrect and premature trials plotted separately. (H) Firing rate patterns for all mPFC cells, sorted by score on PC1–3 and divided into correct, incorrect and premature trials. (I–K) As F and G, analysing cells recorded in the VS.

In order to investigate whether the firing rates of individual neurons in the mPFC or VS were predictive of upcoming premature responses, features were extracted from the firing rate of each neuron (using PCA) in a 1 s window before nose-poke responses for all cells, which were recorded during sessions where rats made more than five premature responses (mPFC, *n* = 784; VS, *n* = 116). These features were then used as independent variables in a logistic regression model to predict upcoming trial outcome (correct or premature responses). Cells with predictive firing rate patterns were identified in the mPFC and VS (236/784 in mPFC, 34/116 in VS) as cells with an AUC of > 0.5. Examples of predictive cells in the mPFC and VS are shown in Fig.3A (mPFC) and Fig.3B (VS), and the population distribution of AUC scores in this window is shown in Fig.3C. The distribution of AUC scores did not differ significantly between the mPFC and VS (Wilcoxon rank sum test, *P* = 0.25), suggesting that there was no difference in the proportion of predictive cells between these regions.

The same analysis was repeated with a moving window across the epoch around the nose-poke event (Fig.3D and E). This analysis indicated that the population mean AUC and proportion of cells with an AUC of > 0.5 peaked when the rat returned to the food magazine on correct trials, peaking slightly later in VS neurons, compared with mPFC neurons.

However, the outcome prediction analysis provided no evidence as to whether there were particular patterns or motifs in firing rate behaviour in mPFC and VS cells, including delay or ramping activity. Therefore, to assess whether firing rate patterns that explained large amounts of the variation in firing rates occurred in the population of mPFC and VS neurons, PCA was applied to the average peri-nose-poke PSTHs of correct trials over the population of cells (Fig.3F–K). The same features were then extracted from incorrect and premature trials. For cells recorded in the mPFC, three PCs explained over 85% of the variance in peri-nose-poke firing rates (PC1-3 explained 43, 22 and 20% of variance, respectively), whereas in the VS, three PCs explained over 81% (PC1-3 explained 37, 26 and 18% of variance respectively, Fig.3F and I).

In both the mPFC and VS, both positive and negative ramp-like activity were major determinants of firing rate, with ramping being represented in PC2 and PC3 in the mPFC and PC1 and PC2 in the VS (Fig.3G and J). In both the mPFC and VS, changes in the firing rate following nose-poking were also represented in the PCs. However, degrees of ramp-like activity appeared to be represented continuously rather than by individual cells with either very strong or completely absent ramp-like activity (Fig.3H and K).

### Ramping neurons in the medial prefrontal cortex and ventral striatum

**FIG. 4:**
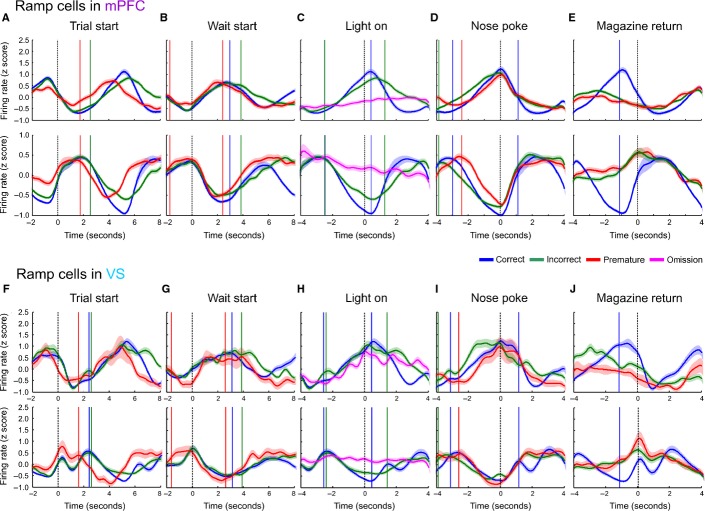
Ramping activity in the mPFC and VS. (A) Mean population PSTH of mPFC cells with positive (top) and negative (bottom) ramping activity aligned to the trial start. (B) Population PSTH of mPFC ramping cells aligned to the wait-start event. (C) Ramping cells in the mPFC aligned to the time of stimulus light presentation (omission trials are shown in purple). (D) Ramping cells in the mPFC aligned to nose-poke event. (E) Ramping cells in the mPFC aligned to magazine return event. (F–J) As A–E, for ramping cells recorded in the VS.

Cells were identified for further analysis as having significant ramping activity as those whose firing rate in the 3 s preceding a correct nose-poke was linear with respect to time with ¦*r*¦ > 0.5, *P* < 0.05 (Bonferonni-corrected to the number of cells), and a ¦β¦-value > 0.3 (to ensure ramping activity rather than consistent flat lines). These criteria therefore did not constrain the time that neurons began their ramping activity, or its end point. In total, 158/897 cells in the mPFC met the criteria for positive ramping and 282/897 met the criteria for negative ramping, whereas in the VS 81/383 and 112/383 cells met the criteria for positive and negative ramping, respectively (Fig.4, Table 2). The proportion of ramping cells did not differ between brain regions (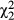 = 2.31, *P* = 0.315, Chi-squared test).

**Table 2:** Total neurons recorded

	mPFC	VS
Total cells firing > 0.5 Hz recorded under standard delay	897	383
Positive ramping cells	158	81
Negative ramping cells	282	112
Non-ramping cells	457	190
Total cells with ≥ 4 prematures recorded under standard delay	784	116
Predictive cells	236	34
Positive ramping cells	137 (54)	23 (10)
Negative ramping cells	244 (80)	34 (11)
Non-ramping cells	403 (102)	59 (13)
Total cells firing > 0.5 Hz under variable delay	211	–
Positive ramping cells	20	–
Negative ramping cells	33	–
Non-ramping cells	158	–

Numbers shown in brackets are the number of predictive cells falling into each subgroup.

Across the population of ramping cells, ramping activity appeared to begin shortly before wait-start, and to reach a maximum (or minimum) at the time of nose-poking (Fig.4). In the mPFC on trials on which rats made premature responses, ramping began earlier relative to the start of the trial (Fig.4A), but relative to nose-poking did not have a steeper gradient or lower maximum (or minimum) compared with correct responses. In the VS, negative ramping activity followed a similar pattern, whereas this was less apparent in positive ramping activity in this structure, although positive ramping activity did reach its pre-ramp minima and post-ramp maxima earlier on premature trials. By contrast, whereas ramping activity began at a similar time for trials ending with correct and incorrect responses, firing rates relative to incorrect nose-pokes (where rats respond after the stimulus presentation but in the incorrect location) exhibited a leftward-shifted firing rate ramp.

In order to confirm that ramping activity was related to active engagement in the task, PSTHs were also plotted relative to the time that the stimulus light was presented, with trials divided into correct, incorrect and omission outcomes. During omission trials, rats did not make a nose-poke response, but the stimulus light was presented (Fig.4C and H). Ramping activity was not present on omission trials in the mPFC and was reduced in the VS, suggesting that this activity was correlated with task engagement.

Given that ramping activity aligned to the wait-start and nose-poke events was similar for both premature and correct nose-pokes, with similar gradients in firing rate increase and similar maxima, these data suggest that, of the proposed models of firing rates, models 3 or 4 best describe ramping activity preceding a premature response. Thus, ramping activity may represent an internal time representation that begins too early on trials that end in a premature response.

### Relationship between behavioural variability and ramping activity

As the 5-CSRTT is a self-paced task, rats are not instructed when to leave the food magazine and start waiting. This potentially confounds averaged PSTH data as trials with different latencies to wait-start are averaged together. Trials with different latencies might have different peak firing rates, or firing rate gradients. Therefore, in order to understand ramping activity during correct and premature responses trials, the time that the rat spent waiting during each trial was analysed.

**FIG. 5:**
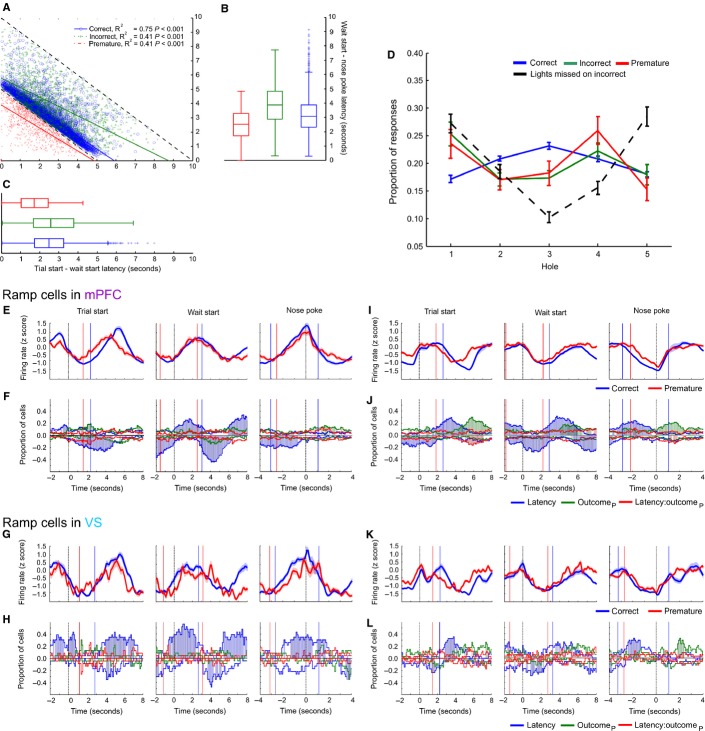
Analysis of 5-CSRTT behavioural variability and ramping activity. (A) Behavioural latencies during 5-CSRTT performance for all behavioural trials across all mPFC and VS rats. Scatter plot of wait-start latency [the time (in seconds) between the start of a trial and the wait-start event (*x* axis)] against wait-start to nose-poke latency (*y* axis) for correct, incorrect and premature trials. (B) Boxplot of wait-start to nose-poke latency for each trial outcome type. (C) Boxplots of wait-start latency for each trial outcome type. (D) Distribution of the location of nose-pokes (expressed as a proportion of all of the responses of the same type over a recording session) for correct, incorrect and premature responses, as well as the location that the stimulus light was illuminated on incorrect trials. (E) Average population PSTH of positive ramping cells recorded in the mPFC aligned to trial start (left), wait-start (middle), and nose-poking (right). (F) Proportions of cells with significant effects of wait-start latency (blue), upcoming premature outcome (outcome_P_, green) or latency × outcome interactions (red) on firing rate. For the effect of latency, positive values indicate a positive correlation between firing rate and latency; for the effect of outcome, positive values indicate higher firing rates on premature trials compared with correct trials. Times when proportions of cells greater than chance were significantly affected by a variable are highlighted with coloured shading. (G) PSTH of population average ramping activity for positive ramping cells recorded in the VS. (H) As F, for positive ramping cells recorded in the VS. (I-L) As E-H, analysing cells with negative ramping activity in the mPFC and VS.

Premature responses were associated with shorter latencies to the start of waiting compared with correct responses [Fig.5A–C, wait-start latencies on premature trials were on average 0.763 ± 0.04 s shorter than on correct trials (mean ± SE), *t*_11408_ = −19.22, *P* < 0.001, linear mixed model], and also with shorter lengths of time spent waiting (latencies from wait-start to nose-poking on premature trials were on average 0.558 ± 0.04 s shorter than on correct trials, *t*_11 408_ = −13.35, *P* < 0.001). Additionally, the wait-start latency correlated negatively with wait-start to nose-poke latency for both correct trials (*F*_1,8818_ = 22659.39, *P* < 0.001) and premature trials (*F*_1,774_ = 851, *P* < 0.001).

The effect of the wait-start latency on firing rate within each trial was directly measured using a GLM for the firing rate of each cell, using both upcoming trial outcome and wait-start latency as independent variables. In order to provide an accurate comparison between correct and premature trials, cells recorded during sessions where fewer than four premature responses were made were excluded from analysis (see Table 2 for total numbers of cells analysed). Across the population of ramping cells recorded in both the mPFC and VS, there were no epochs between wait-start and nose-poking where significant proportions of ramping neurons (either positive and negative) differentiated between correct and premature responses, or where there were significant interactions between the wait-start latency and upcoming outcome (Fig.5F, H, J and L). Averaged firing rates in the same epoch are illustrated in Fig.5E, G, I and K. There were, however, large populations of cells whose instantaneous firing rate correlated with the wait-start latency.

Relative to the start of the trial for cells recorded in the mPFC, premature trials began ramping significantly earlier than correct trials, in keeping with the behavioural data (Fig.5E, G, I and K). For the PSTH aligned to the start of the trial there were time bins with significant proportions of cells with a negative correlation between firing rate and wait-start latency, a significant, positively valued effect of upcoming trial outcome and a significant, negatively valued interaction between firing rate and ramping around the median wait-start latency for correct trials. For positive ramping cells, a signficant population of neurons exhibited a negative correlation with wait-start latency preceding nose-pokes; trials with longer wait-start latencies were accompanied by more rapid increases in firing rate towards the time of nose-poking compared with those trials with a short wait-start latency (where the rat spent longer waiting and therefore suggesting that the firing rate increased more gradually). The same pattern of response was observed for negative ramping cells, but with the sign of the effect inverted. Similar patterns of activity were also present in the VS, but with fewer recorded cells with both correct and premature responses this analysis carried less power to detect significant effects.

In order to investigate whether premature responses differed from correct responses, the locations of correct, premature and incorrect nose-pokes, as well as the stimulus light presentations missed on incorrect trials were analysed (Fig.5D). The proportion of nose-pokes in each of the five holes was significantly different for different trial outcomes (interaction between location and trial outcome, *F*_8,1620_ = 4.104, *P* < 0.001, linear mixed model). Importantly, whereas the distribution of correct responses differed from those of incorrect and premature responses (*t*_1620_ = 2.592, *P* = 0.010 and *t*_1620_ = 2.145, *P* = 0.032 respectively), the distribution of incorrect and premature responses did not differ (*t*_1620_ = 0.379, *P* = 0.705).

Within each outcome type, correct nose-pokes were significantly less likely in the four peripheral holes compared with the central hole (all *t* < 0, *P* < 0.05). For comparison, on incorrect trials, the missed stimulus light was significantly more likely to be in a peripheral hole compared with the central hole (all *t* > 0, *P* < 0.05). The situation was more complex for incorrect and premature responses with nose-pokes being significantly more likely in holes 1 and 4 compared with hole 3 (the central hole) for incorrect responses (all *t* > 0, *P* < 0.05, with no difference in the proportion of responses between hole 3 and holes 2 or 5, all *P* > 0.05). Premature responses were significantly more likely in hole 4 compared with hole 3 (*t*_432_ = 2.332, *P* = 0.020), but there was no difference in the proportion of responses between hole 3 and holes 1, 2 or 5 (all *P* > 0.1). These results demonstrate that the distributions of incorrect and premature responses were similar, and both differed from correct responses, suggesting that, whereas ramping activity (once initiated) may be similar on premature and correct trials, the behavioural responses selected differ between these trial types.

### Effects of variable waiting periods on ramping activity in the medial prefrontal cortex

Neurons in the mPFC have been described as tracking behavioural strategies, encoding switches between rules used to guide behaviour (Rich & Shapiro, 2009). The preceding analyses have demonstrated that one feature of mPFC and VS neurons during 5-CSRTT performance is ramping activity, which appears to peak at the point that rats make a nose-poke response. However, in the standard implementation of the 5-CSRTT there is only one delay length presented (5 s), so it is ambiguous as to whether the delay-related activity peaks at the time that the rat anticipates the stimulus light occurring or rather a ‘deadline’ for the time that a response should be made, as predicted by some models of reaction time task performance (Ollman & Billington, 1972).

**FIG. 6:**
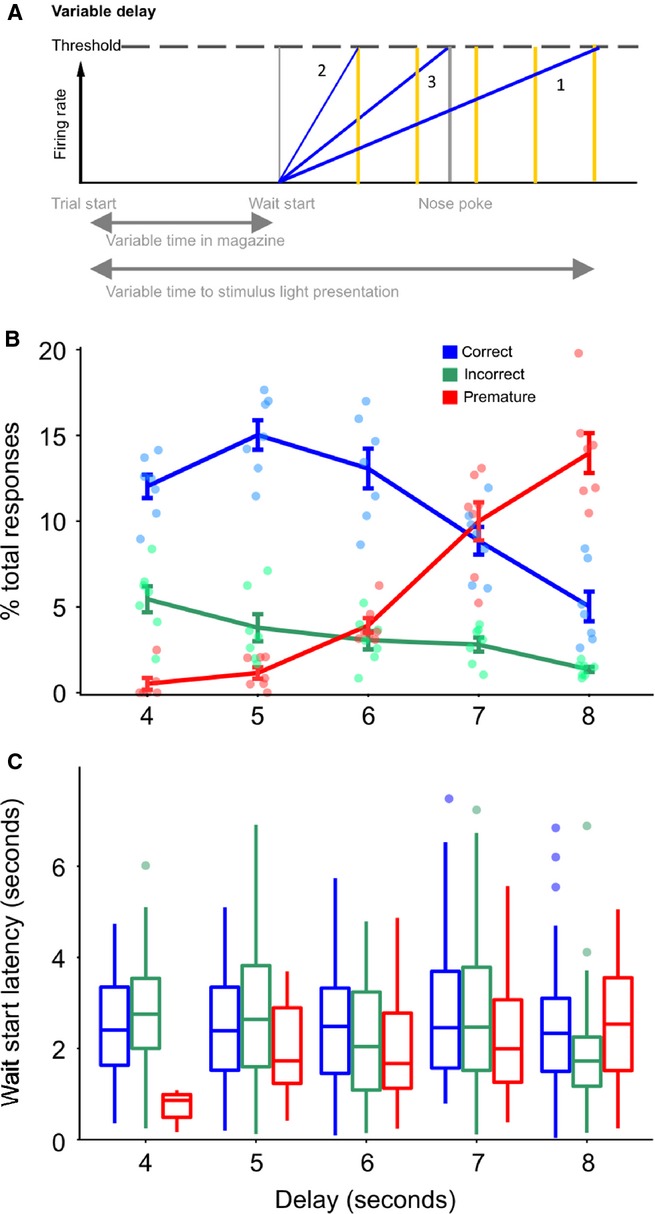
Effect of variable delay intervals on behaviour. (A) Models for the effect of variable delay on ramping activity. Model 1 – activity ramps towards the latest possible time that the stimulus could be presented. Model 2 – activity ramps towards the earliest possible time that the stimulus could be presented. Model 3 – activity ramps towards the time of the nose-poke response. The possible times of the stimulus light are indicated as vertical yellow lines. (B) Observed behavioural effects of variable delay; proportion of responses at each of the offered delays. (C) Wait-start latency [the average time (in seconds) between the start of a trial and the wait-start event] at each of the offered delay periods.

We hypothesized that, if the delay is made variable, and if the deadline model describes ramping activity, delay activity should reach its maximum at the longest possible delay, with delay activity consequently being lower at nose-poking for trials with shorter delays. Alternatively, ramping activity could peak either at the earliest time that a stimulus is presented, representing the time when the rat most closely attends to the stimulus lights, or when the rat makes a nose-poke response regardless of the distribution of possible delays (Fig.6A). We tested these predictions by randomly varying the delay between 4, 5, 6, 7 or 8 s. Single units were recorded in the mPFC in seven rats during a single session where the delay was made variable (variable delay sessions). Behaviourally (Fig.6B and C), the proportion of premature responses increased as the delay length increased during variable delay sessions (effect of delay, *F*_4,25_ = 57.04, *P* < 0.001, linear mixed model). Relative to the 4 s delay trials, the proportion of correct trials increased for 5 s delay trials (*t*_25_ = 2.38, *P* = 0.025), was no different on 6 s delay trials (*t*_25_ = 0.822, *P* = 0.042), and significantly decreased on 7 s delay trials (*t*_25_ = −2.52, *P* = 0.018) and 8 s delay trials (*t*_25_ = −5.57, *P* < 0.001). Incorrect responses also decreased as the delay period increased (*F*_4,25_ = 6.61, *P* = 0.001).

However, the delay length did not affect the wait-start latencies for correct (*F*_4,610_ = 2.186, *P* = 0.069) or incorrect (*F*_4,184_ = 1.513, *P* = 0.200) trials, but the wait-start latency did differ between delays on premature trials. Relative to trials with a delay of 8 s, trials with a delay of 6 and 4 s had shorter latencies to wait-start (*t*_327_ = −2.98, *P* = 0.003 and *t*_327_ = −2.65, *P* = 0.009, respectively).

**FIG. 7:**
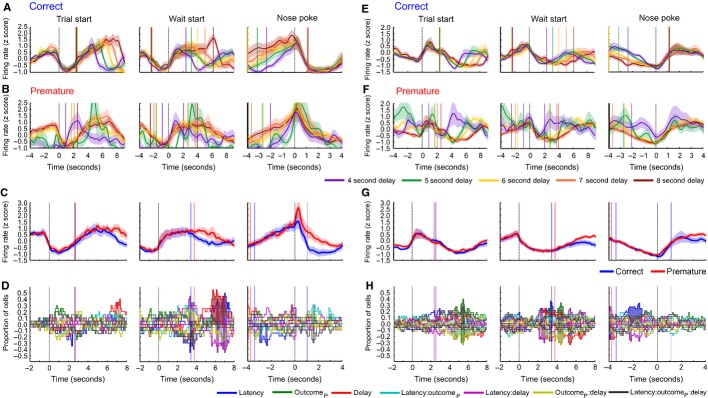
Population activity of ramping cells in the mPFC recorded during variable delay sessions. (A) Population activity around three alignment events (from left to right trial start, wait-start and nose-poking) for trials ending in correct responses recorded in positive ramping cells. Responses are divided by the delay of the trial (represented as a rainbow colour scale). The median time of the next, or previous behaviour event is indicated as a vertical line in the same colour as the firing rate for each delay. (B) As A, for premature responses in positive ramping cells. (C) PSTH of population average ramping activity for positive ramping cells (averaged over all delays), aligned to trial start (left), wait-start (middle), and nose-poking (right). (D) Proportions of cells with significant effects of each independent variable (see colour key for the colour of each variable, where outcome_P_ represents premature outcome) and firing rate. (E–H) As A–D, for negative ramping cells.

A total of 211 cells were recorded in the mPFC during the variable delay sessions. Of these cells, 20 met the criteria for positive ramping, whereas 33 met the criteria for negative ramping. These proportions were significantly different to those observed in the mPFC under standard delay conditions (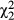 = 39.7, *P* < 0.001, Chi-squared test). As occurred under standard delay conditions, ramping began at the start of waiting, reaching a maximum (or minimum) preceeding the earliest time of stimulus presentation, and remained altered until the time of nose-poking (Fig.7A, B, E and F). Similar patterns were observed during premature trials. The effect of the variable delay session on ramping activity was quantified by fitting a GLM to the firing rate of each cell in successive peri-event time bins, as before, with upcoming trial outcome, wait-start latency and trial delay as independent variables (Fig.7D and H). As observed under standard delay conditions, there were no pre-nose-poke effects of upcoming trial outcome, or trial delay. These results therefore suggest that delay activity in the mPFC ramps towards the estimated earliest time of stimulus presentation, perhaps therefore indicating the time at which attention must be maximally directed towards stimulus detection.

## Discussion

The neurophysiological properties of single units recorded in the mPFC (*n* = 897) and VS (*n* = 383) were analysed during sustained attentional performance. Neural activity was recorded during sessions on which the visual target stimuli were presented after fixed or variable delays and showed a wide range of firing rate patterns, including outcome-related changes in firing rate following nose-poke responses (> 50% of cells in both the mPFC and VS), and delay-related ramping activity that arose as rats first engaged in waiting behaviour (as measured by the onset of behaviour where rats scanned the response apertures) and persisted until a nose-poke response was made (49.1% of cells in the mPFC and 50.4% of cells in the VS). Intriguingly, on trials that ended prematurely, ramping activity commenced earlier but reached the same peak rate at the time of responding. A subpopulation of cells exhibited firing rate patterns that could be used to predict upcoming premature responses (30.1% of cells in the mPFC and 29.3% of cells in the VS) but, at a population level, these predictive cells did not show a consistent pattern of firing. Rather, individual neurons in the mPFC and VS appeared to mainly encode trial outcome after a nose-poke response had been made.

Ramping neural activity was observed in the mPFC and VS during the waiting period, reaching a maximum (or minimum) at the time of nose-poking. Importantly, this activity also occurred on trials ending in a premature response and did not reach a lower peak or have a steeper gradient on these trials. However, the onset of waiting behaviour, and ramping firing activity, did begin earlier on premature trials, and notably reached a peak level at times when nose-pokes were made, which on premature trials were by definition in advance of the presentation of the light stimulus.

Although our GLM results provide support for model 3 (that ramping activity began earlier on premature trials, but had the same gradient as correct trials), our behavioural results demonstrated that premature responses were associated with both shorter latencies to wait-start and shorter waiting times before nose-poking. This would therefore support model 4. It is possible, however, that the small average difference in latencies between correct and premature trials (< 1 s) and the small number of premature trials in the dataset meant that small differences in firing rate gradient between correct and premature trials could not be detected by the current analysis. Despite this limitation, these results demonstrate that premature responses are not associated with either a lower peak (model 1) or a significantly steeper gradient (model 2) of ramping activity than correct trials. Additionally, we have previously demonstrated (Donnelly *et al*., 2014) that wait-start latencies can be used to make informative predictions about the upcoming outcome of a trial. Therefore, if the onset of ramping activity is determined by the start of waiting behaviour, and the gradient of ramping is determined by the length of time spent before waiting begins, given the differences in wait-start latency between correct and premature trials, additional differences in ramping activity between correct and premature trials may be unlikely.

In reaction time tasks such as those described by Ollman & Billington (1972), with a variable delay period, or in the human version of the 5-CSRTT where faster responses obtain greater monetary reward (Voon *et al*., 2014) it is economically favourable for the subject to respond as fast as possible. Combined with a degree of uncertainty in the delay on such tasks it is possible that a falsely detected stimulus might trigger a premature response. However, in the 5-CSRTT there is a fixed, well-trained delay, five possible behavioural responses, and a long opportunity to respond (i.e. the limited hold period of 5 s) with a low probability of a premature response. Consequently, we also investigated the effects of variable pre-stimulus delays on neural activity associated with the various trial types on the 5-CSRTT. As well as increasing premature responses during the longer delays of 6, 7 and 8 s we found that delay-related activity reached a maximum at the time of the earliest possible stimulus presentation and remained altered until the time of the nose-poke response. This suggests that the peak of ramping activity during 5-CSRTT performance may reflect either the onset of the time when a stimulus may be expected or a signal when to selectively deploy attention to the response apertures. Such ramping could also signal the times when maximum action restraint is required or conversely when the subject should prepare to move.

Given the relative paucity of predictive cells found in this study in the mPFC or VS, the neural locus of the decision to make a premature response on this task is unclear. Previous studies have found no effect of neurotoxic or reversible lesions of the pre-limbic region of the dorsal mPFC on premature responding (Chudasama & Muir, 2001; Murphy *et al*., 2012). Moreover, electrophysiological recordings during a T-maze task found that signals related to upcoming actions were not present in the dorsal mPFC, but did occur in the supplementary motor cortex (Sul *et al*., 2010). Recent evidence also suggests that cells in the supplementary motor cortex are predictive of an upcoming termination of waiting (Murakami *et al*., 2014). However, outcome predictive cells have previously been described in the dorsal mPFC during performance of a simple reaction time task and both reversible inhibition and antagonism of dopamine D1 receptors in this region have been reported to increase premature responding and shorten time estimation (Narayanan & Laubach, 2006; Narayanan *et al*., 2006; Parker *et al*., 2014). There is therefore mixed evidence for a role of the dorsal mPFC in regulating waiting behaviour, which could reflect differences in the tasks used in different studies, particularly given the importance of sustained visual attention in 5-CSRTT performance, in addition to time estimation.

One model of VS function suggests a role of this region in maintaining responding over long delays (Nicola, 2006). VS lesions do not cause marked increases in premature responding on the 5-CSRTT (Christakou *et al*., 2004) but do impair performance on the differential reinforcement of low rates of responding (DRL) task where delays are typically longer and uncued (Pothuizen *et al*., 2005; Fletcher *et al*., 2009). Therefore, it is possible that the VS may be preferentially activated as delays are increased. Thus, electrophysiological correlates of a failure to wait might only be observed in the VS over longer delays.

The mPFC and VS may instead have modulatory roles in 5-CSRTT performance, signalling the time elapsed, as well as the outcome of behaviour after it has been performed. We demonstrated that LFP oscillations in these brain regions were also influenced by the recent reward history of the rat during 5-CSRTT performance (Donnelly *et al*., 2014), further reinforcing the view that multiple sources of information on behavioural performance are integrated in the mPFC and VS. However, the effect of previous reward history could not be directly measured in the current study as relatively few premature responses were made during the recording sessions. This proportion would have been even smaller had premature responses been divided into previously rewarded and non-rewarded responses, thereby reducing the power of the analysis. Nevertheless, as we previously reported that the wait-start latency was influenced by previous trial reward history (Donnelly *et al*., 2014), the behavioural consequences of previous trial outcomes have been included in the present analysis.

Ramping or delay activity has been observed in brain structures other than the mPFC and VS, including in the dopaminergic ventral tegmental area (Totah *et al*., 2013) and serotonergic dorsal raphé nucleus (DRN) (Miyazaki *et al*., 2011a), regions that have reciprocal connections with the mPFC and VS (Maurin *et al*., 1999; Carr & Sesack, 2000; Joel & Weiner, 2000; Watabe-Uchida *et al*., 2012). In the DRN, the firing rate of 5-HT neurons in the DRN increased during a waiting period, but decreased in advance of a premature response. Moreover, pharmacological inactivation of the DRN increased premature errors, whereas optogenetic activation of the DRN reduced premature terminations of waiting (Miyazaki *et al*., 2011b, 2012, 2014). These data therefore suggest that the DRN 5-HT system may be causally involved in waiting behaviour.

Pre-stimulus ramping activity has also been described in the ventral tegmental area (Totah *et al*., 2013), and it has also been demonstrated in a time estimation task that ramping single unit activity and waiting-related low-frequency LFP oscillations are disrupted by antagonism of dopamine D1 receptors in the mPFC (Parker *et al*., 2014), suggesting that the ventral tegmental area dopaminergic projection to the mPFC is also involved in waiting or timing behaviour. In the case of the 5-CSRTT, whereas dopamine D1 receptor agonism in the pre-limbic mPFC has been shown to increase attentional accuracy (Granon *et al*., 2000), increasing mPFC dopamine through inhibition of its reuptake has not been shown to alter premature responding (Economidou *et al*., 2012), so the precise role of mPFC dopamine in this task requires further investigation.

In contrast with the present study, the firing of neurons relative to the time of behavioural responses in both the DRN and ventral tegmental area has been reported to differ in advance of correct and premature responses (Miyazaki *et al*., 2011a; Totah *et al*., 2013). There are several possible explanations for this discrepancy. Firstly, as described above it is possible that the relatively short delays used in the present study were not sufficiently long to place high demands on action restraint, which may preferentially recruit VS activity.

Alternatively, it is possible that premature responses in our implementation of the 5-CSRTT do not result from a failure of a waiting process and equally, incorrect responses may not result from a failure to deploy attentional resources at the time of stimulus presentation. Perhaps instead, under standard task conditions, where rats have been extensively trained over many weeks, rats begin waiting on a trial and, in addition to attending to the response apertures (‘scanning’ behaviour), rats utilize a representation of time in the mPFC and VS to focus attention at the expected onset of the stimulus. If the rats have not correctly detected the stimulus light when ramping activity reaches its peak, either due to early onset of waiting, or due to failures in deploying visual attention to the nose-poke apertures, this information may then increase the likelihood of rats making a riskier ‘guess’ response to one of the nose-poke apertures. Premature responses might occur under these circumstances not because of a failure to wait *per se* but because the timing process begins too early, combined with a failure to use visual cues to guide behaviour. This hypothesis would explain the failure to find any major pre-response correlates of upcoming incorrect or premature responses and the finding that premature and incorrect responses were made in similar locations. Additionally, this view is consistent with our recent observation that behavioural measures, especially the wait-start latency, provide the best predictors of impending premature responses rather than neurophysiological signals in the mPFC and VS (Donnelly *et al*., 2014).

In conclusion, the main findings of this investigation show that, in a behavioural task involving waiting and selective visual attention, neurons in the mPFC and VS respond to rewards and errors as well as delays. This delay-related activity did not substantially differ between correct and premature trials but began earlier when rats made premature responses. When the delay period was made variable, delay activity ramped up (or down) to the earliest possible time of stimulus presentation and remained altered until a nose-poke response was made. These findings therefore suggest that premature responses may not result from a failure in a waiting process but instead from rats incorrectly timing the delay to the expected time of stimulus presentation. Our findings imply that the neural locus of the decision of when (and where) to make a premature response may emanate from structures outside the mPFC and VS. Based on recent empirical studies this integration may occur in the supplementary motor cortex (Murakami *et al*., 2014) and other structures implicated in impulsivity, including monoaminergic systems in the midbrain. Such research may be relevant to understanding the aetiological basis of maladaptive impulsivity in attention-deficit hyperactivity disorder and drug addiction.

## Abbreviations

5-CSRTT: five-choice serial reaction time task
AUC: area under the receiver-operator characteristic curve
DRN: dorsal raphé nucleus
GLM: general linear model
mPFC: medial prefrontal cortex
PC: principal component
PCA: principal components analysis
PSTH: peri-stimulus spike rate histogram
VS: ventral striatum
